# Rice Cultivation Area, Demographic Trends, and Trade Dynamics for Food Security in Nepal (2011–2021)

**DOI:** 10.1002/pei3.70020

**Published:** 2024-12-06

**Authors:** Nabin Lamichhane, Urmila Dhami, Durga Dhakal, Lal Bahadur Thapa

**Affiliations:** ^1^ Central Department of Botany Tribhuvan University Kirtipur Kathmandu Nepal; ^2^ Dhading Polytechnic Institute Council for Technical and Vocational Education and Training (CTEVT) Dhading Nepal

**Keywords:** cultivation, food security, import dependency, import/export, population dynamics, production

## Abstract

Rice is the most important staple crop in Nepal, playing a critical role in both the economy and food security. This study analyzes the trends in rice cultivation, production, imports, and exports from fiscal years 2011/2012 to 2021/2022 and also presents population data from the initial and final years. Over the study period, the area of rice cultivation declined by 0.81% annually, while the production grew by 1.5% per year, and the yield improved at a rate of 1.97% per year. Trend analysis indicated no significant changes in cultivation area or production, but a significant positive trend was observed in the yield. Rice import showed a significant annual increase of 5.61% in price value and 12.80% in quantity, while exports also grew by 1.95% in quantity and 2.39% in value. However, exports remain negligible compared to imports. Nepal's rice self‐sufficiency ratio (SSR) has declined by 1.15% annually, falling from 92.72% in 2011/2012 to 82.01% in 2021/2022 while its import dependency ratio (IDR) has increased by 5.89% annually. These trends suggest that Nepal is becoming increasingly vulnerable in terms of rice food security. Population dynamics based on two census records revealed a notable 14% rise in the foreign population. This situation underscores the urgent need for policy interventions to address the decline in rice self‐sufficiency, labor shortages, and growing import dependency, ensuring sustainable rice production and food security in Nepal.

## 1 Introduction

Rice is a fundamental staple crop globally and is particularly significant in South Asia (Fukagawa and Ziska 2019), where it plays a key role in regional economies and food security. In Nepal, rice stands out as the most important staple crop, contributing substantially to the country's food security (Dahal, Ghimire, and Poudel 2022). A total of 2,680,955 households in Nepal are engaged in mainland rice farming. When upland rice and early rice farming are also included, 53.67% of households in the country are directly involved in rice cultivation (NSO 2023). The average per capita consumption of rice is 174 kg/year (Dhungel and Acharya 2017), making it the most important food source in the Nepali diet. This crop not only provides 69% of the daily caloric intake of the population (Dhungel and Acharya 2017) but also contributes around 20% of Nepal's agricultural GDP (CDD 2015; Dhungel, Mahat, and Pant 2024), underscoring its vital role in the Nepalese economy. So its availability and affordability are directly linked to the population's well‐being (Gartaula et al. 2017).

Geographically, rice cultivation spans a wide range of altitudes from 67 m in the Tarai to 3050 masl in high hills in Nepal (Gadal et al. 2019). The districts like Morang, Chitwan, Jhapa, Sunsari, and Rupandehi are major contributors to national production in Nepal, though rice is cultivated throughout all the districts to some extent except Manang and Mustang (MoALD 2022). Despite this, Nepal faces productivity challenges, with yields below potential due to poor access to quality seeds, limited irrigation, and insufficient use of modern farming techniques (Choudhary et al. 2022; Bhandari et al. 2023; Nepal et al. 2024). The challenge is exacerbated by climate change, for example, erratic rainfall patterns (Rayamajhee, Guo, and Bohara 2021). As a result, Nepal's growing dependence on rice imports to meet domestic demand presents a significant food security concern.

Considering the trends in rice cultivation, production, yield, and import/export is crucial for understanding the sustainability of food security in Nepal (Gumma et al. 2011; Poudel and Chen 2012). As rice is the most important staple food, these factors directly affect the nation's ability to feed its population (Dhungel and Acharya 2017; Tiwari et al. 2023). Monitoring cultivation trends reveals whether Nepal's agricultural land use is sufficient to meet growing demands, while production and yield data reflect the efficiency and capacity of rice farming systems under changing climatic and socioeconomic conditions (Choudhary et al. 2022). A decline in cultivation or stagnation in yield signal vulnerabilities in domestic food production, necessitating timely interventions (Bishwajit et al. 2013). Furthermore, tracking import and export trends is essential for understanding Nepal's dependency on foreign rice supplies (Khanal 2023). A rising import trend combined with a shrinking self‐sufficiency ratio (SSR) increases the country's vulnerability to global market fluctuations and trade disruptions, which can lead to food insecurity (Timsina et al. 2023). On the other hand, an increase in rice exports could signal the potential for economic growth, but only if it does not undermine the domestic food supply (Sah et al. 2024). Given Nepal's challenges with labor migration, climate variability, and limited agricultural infrastructure, analyzing these trends helps policymakers design informed strategies to enhance local rice production, reduce dependency on imports, and safeguard national food security (Bhusal 2023). The stability of rice production directly impacts the country's gross domestic product (GDP) and overall economic well‐being, as any fluctuations can lead to increased import expenditures, straining economic resources and affecting the agricultural workforce, which forms the backbone of Nepal's economy (Gairhe, Gauchan, and Timsina 2021; Choudhary et al. 2022; Timsina et al. 2023). Ensuring a stable and self‐sufficient rice production system is essential for economic stability and sustainable growth, especially considering the increasing demand, changing climate conditions, and the country's vulnerability to external factors (Tiwari et al. 2023).

The self‐sufficiency ratio (SSR) and import dependency ratio (IDR) are critical indicators for understanding a country's rice food security (Pokhrel 2020; Niu et al. 2022; Prayuginingsih et al. 2022). The SSR measures the percentage of rice produced domestically relative to national consumption needs (Lim 2024), with a higher SSR indicating greater self‐reliance and resilience in the face of global market fluctuations. Conversely, a declining SSR signifies increasing reliance on imports, potentially putting the country at risk of food insecurity during global supply disruptions (Kaufmann et al. 2022). The IDR quantifies the extent of a nation's dependency on imported rice, with a rising IDR signaling a vulnerability to external factors such as international price volatility, trade barriers, or currency fluctuations, all of which can destabilize access to affordable rice (FAO 2011). In Nepal, these indicators are directly tied to the country's ability to maintain food security (Pokhrel 2020). Additionally, farm population plays a crucial role in understanding the workforce available for agricultural production. A significant portion of Nepal's adult population is involved in agriculture, and demographic shifts such as labor migration to foreign countries or urbanization can reduce the agricultural labor force (Pant 2013), decreasing rice production and lowering the SSR. As more working‐age individuals migrate, domestic rice farming becomes increasingly constrained, exacerbating the country's reliance on imports (Jaquet et al. 2015; Gauchan and Shrestha 2017). Thus, linking SSR and IDR with census data offers a comprehensive understanding of how demographic changes and labor dynamics affect agricultural productivity, import dependency, and overall food security. Policymakers can utilize these insights to address labor shortages, enhance domestic rice production, and mitigate growing import reliance, ultimately working towards strengthening Nepal's food security.

This study addresses the following questions: (i) What is the current state of rice cultivation in Nepal, and how has it changed between the years 2011/2012 and 2021/2022? (ii) What are the dynamics of the rice trade in Nepal? (iii) How have the rice self‐sufficiency ratio (RSSR) and import dependency ratio (IDR) changed over the past decade, and what does this indicate about the direction of rice food security in Nepal? (iv) How have the active, foreign, and farming populations changed between 2011 and 2022? To answer these questions, secondary data from the relevant ministries and departments of the Government of Nepal were analyzed. The findings could be helpful to provide valuable insights for policy recommendations and management strategies to enhance food security in the region.

## 2 Materials and Methods

### 2.1 Data Collection and Analysis

This study analyzed secondary data to examine the dynamics of rice cultivation, production, population, and food security in Nepal over a decade (2011–2021). Rice cultivation, production, and yield data for fiscal years 2011/2012 to 2021/2022 were obtained from the “Statistical Information of Nepalese Agriculture” published by the Ministry of Agricultural and Livestock Development, Government of Nepal (MoALD, https://moald.gov.np/). A fiscal year (FY) is a 12‐month period for financial reporting. Nepal's FY runs from mid‐July to next mid‐July, typically from Shrawan 1 (around July 16) to Ashad 31 (around July 15). Rice import and export data were retrieved from the “Nepal Foreign Trade Statistics” provided by the Department of Customs, Ministry of Finance, Government of Nepal (https://www.customs.gov.np/).

RSSR and IDR are important indicators used to assess a country's food security, particularly in terms of rice (Bishwajit et al. 2013; Li et al. 2024). The RSSR measures the extent to which a country can meet its rice consumption needs from domestic production. It is calculated as follows (FAO 2011):

$$\text{RSSR}=\frac{\text{Domestic rice production}}{\text{Total rice consumption}}\times 100.$$

The IDR measures the proportion of a country's rice consumption that is satisfied through imports. It is calculated as follows (FAO 2011):

$$\mathrm{IDR}=\frac{\text{Import}}{\text{Production}+\text{Import}-\text{Export}}\times 100.$$

Demographic data, including total population, active population (15–59 age group), and individuals working abroad, were obtained from the Census Reports of Nepal (CBS 2011, 2021, https://censusnepal.cbs.gov.np/), published by the Central Bureau of Statistics Nepal. Population dynamics were assessed by calculating percentage changes in total population, active population, and foreign workers.

### 2.2 Statistical Analysis

Trend analysis was conducted on the data from the fiscal years 2011/2012 to 2021/2022. The analysis included area, production, and import trends using data from 11 years. The export data for 2014/2015 was missing, and the data for 2012/2013 was considered as an outlier and excluded from the analysis. Therefore, for the export data, rice import dependency ratio, and rice self‐sufficiency ratio, only 9 years of data were included.

The trends in rice dynamics and trade were assessed using the Mann–Kendall trend test followed by Sen's Slope calculation to estimate the magnitude of the change (Ali and Abubaker 2019). The rate of change (%) per annum for all the parameters was calculated using the formula:

$$\text{Rate of change}\;\left(\%\right)\;\mathrm{per}\;\text{annum}=\frac{{\mathrm{Sen}}^{\prime }s\;\text{Slope}}{\text{Mean}}\times 100\%.$$

Mann–Kendall trend statistics were computed using the *mk.test* function, and Sen's Slope was calculated using the *sens.slope* function from the *trend* package in R.

To elucidate the relationship between the rice self‐sufficiency ratio and import dependency ratio with other measured parameters, principal component analysis (PCA) was performed using the *FactoMineR* package (Lê, Josse, and Husson 2008). All statistical analyses were conducted in R (R Core Team 2024).

## 3 Results

### 3.1 Rice Cultivation Area

**FIGURE 1:**
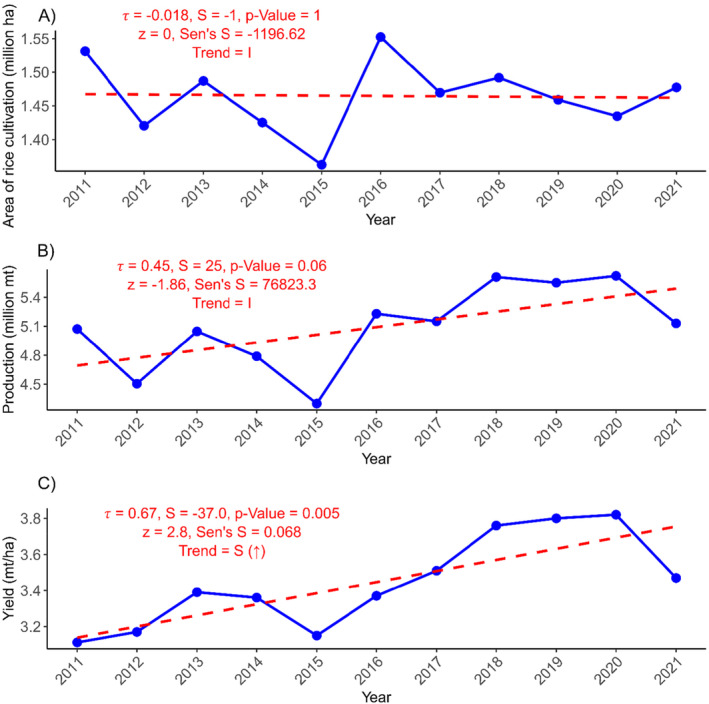
Trends in rice cultivation: (A) Rice cultivation area; (B) rice production; (C) yield. Mt, metric tons; *S*, Mann–Kendall's statistics; *τ*, rank correlation coefficient; *z*, standardized test statistic; Sen's *S*, Sen's slope; *S* (↑), significant increase; *S* (↓), significant decrease; *I*, insignificant. Fiscal years are represented by the starting year; for example, 2011/12 is shown as 2011.

The area under rice cultivation in Nepal decreased from 1,531,493 ha (ha) in 2011 to 1,477,378 ha in 2021 (Figure 1A). The cultivation area was declined by 0.81% annually; however, the trend was not significant (*τ* = −0.018, *z* = 0, *p* = 1) (Figure 1A).

### 3.2 Rice Production and Yield

The mean production of rice over the 11 years was found to be 5,091,542 mt while the mean yield was found to be 3.44 mt/ha. The rate of change in rice yield is 1.97% per annum, and the rate of change in the production is 1.50% per annum (Table 1). The trend analysis showed that yield increased significantly (*τ* = 0.67, *z* = 2.8, *p* = 0.005); however, the production result shows an insignificant trend (*τ* = 0.45, *z* = −1.86, *p* = 1) (Figure 1).

**TABLE 1:** Percentage change per annum for parameters from 2011/2012 to 2021/2022 based on Sen's slope analysis.

Parameters	Rate of change per annum (%)
Rice cultivation area (ha)	−0.81
Production (mt)	1.50
Yield (mt/ha)	1.97
Import quantity (mt)	5.61
Import value (NRs)	12.80
Export quantity(mt)	1.9
Export value (NRs)	2.39
Rice self‐sufficiency ratio	−1.15
Import dependency ratio	5.89

Abbreviations: Mt, metric tons; NRs, Nepalese rupees.

### 3.3 Rice Import and Export

**FIGURE 2:**
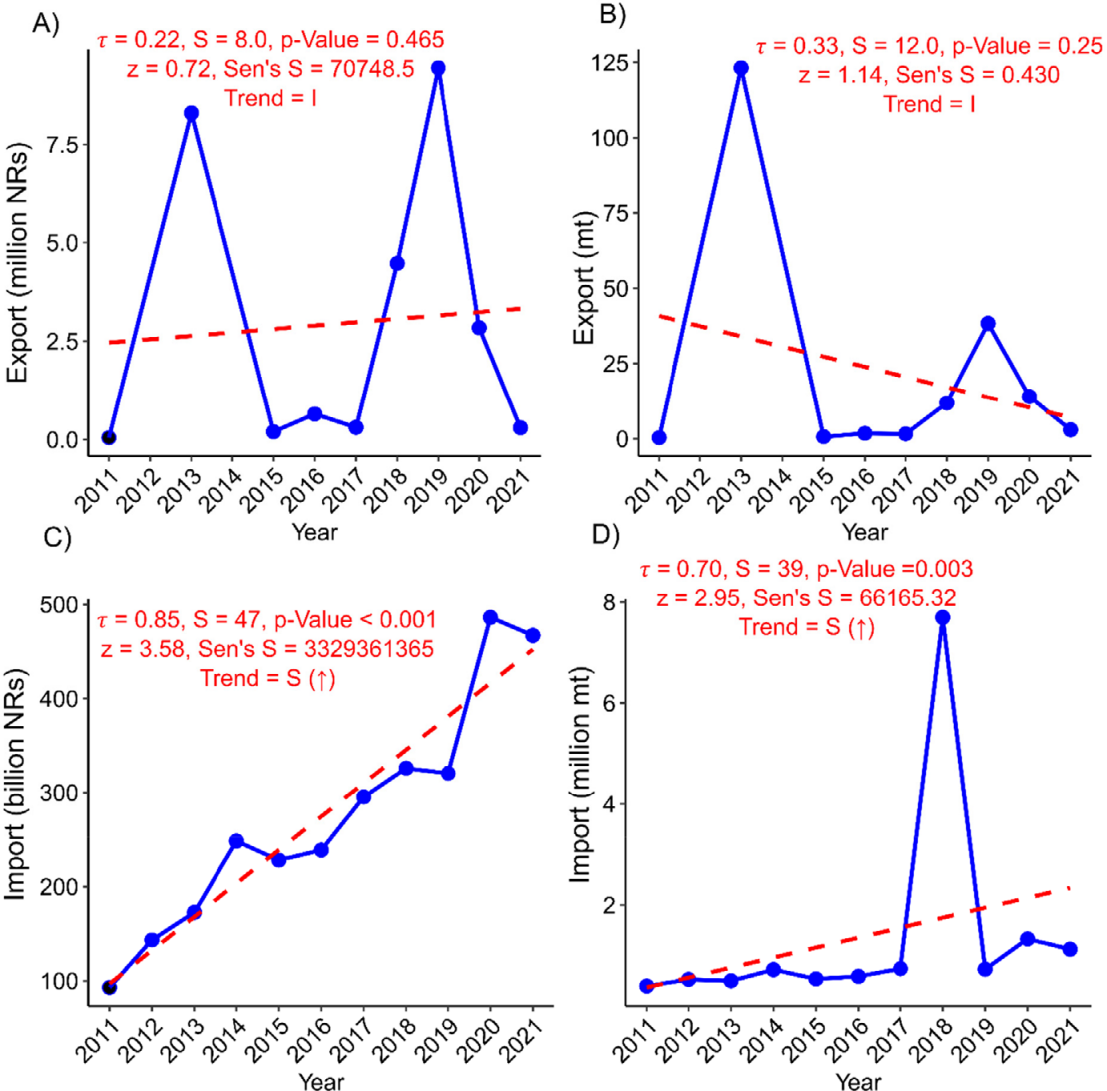
Rice trade dynamics: (A) Export economic value; (B) export quantity; (C) import economic value; (D) import. mt, metric tons; NRs, Nepali rupees; S, Mann–Kendall's statistics; *τ*, rank correlation coefficient; *z*, standardized test statistic; Sen's *S*, Sen's slope; *S* (↑), significant increase; *S* (↓), significant decrease; *I*, insignificant. Fiscal years are represented by the starting year; for example, 2011/2012 is shown as 2011.

**FIGURE 3:**
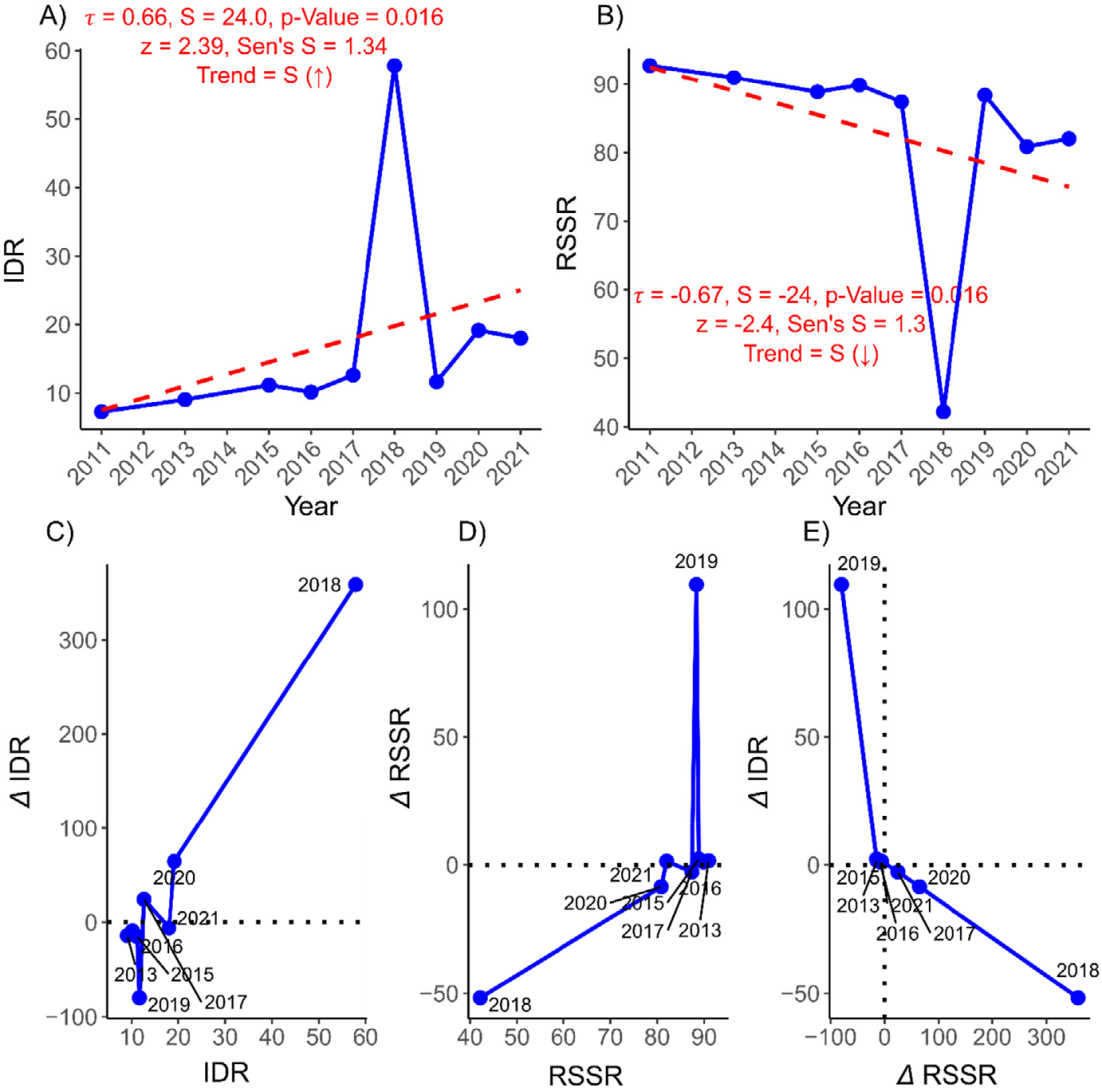
Trend in rice security status: (A) Rice import dependency ratio; (B) rice self‐sufficiency ratio; (C–E) rice import dependency ratio and rice self‐sufficiency ratio variation in different years. RSSR, rice self‐sufficiency ratio; IDR, rice import dependency ratio; ∆IDR, rate of change (annual) in rice import dependency ratio; ∆RSSR, rate of change (annual) in rice self‐sufficiency ratio metric tons; *S*, Mann–Kendall's statistics; *τ*, rank correlation coefficient; *z*, standardized test statistic; Sen's *S*, Sen's slope; *S* (↑), significant increase; *S* (↓), significant decrease; *I*, insignificant. Fiscal years are represented by the starting year; for example, 2011/2012 is shown as 2011.

The export of rice is very low as compared to import (Figure 2A–D). The rate of change in rice import was found to be 5.61% per annum, while monetary value was 12.80% (Table 1). In 2011, Nepal imported 398,483 metric tons of rice, but by 2021 this figure increased over 647,961 metric tons (Figure 2D). This surge in imports significantly increased in expenditure, rising from NRs. 92.88 billion in 2011 to NRs. 467.03 billion in 2021 (Figure 2C). Similarly, the rate of change in the export was 1.9% in terms of quantity and 2.39% in terms of monetary value (Table 1). The trend analysis showed that both the price of rice imports and quantity were increased significantly (*p* < 0.001, 0.003 respectively, Figure 2). However, the export trend is insignificant and remains negligible compared with imports (Figure 2). As a result, Nepal's reliance on imported rice has increased from 7.28% to 11.21%, as shown by IDR, while RSSR has decreased from 92.72% to 88.89% (Figure 3, Table S1). The rice IDR is increasing by 5.89% annually, whereas RSSR is decreasing by 1.56%. This trend of increasing dependency and decreasing rice self‐sufficiency is significant (Figure 3).

**FIGURE 4:**
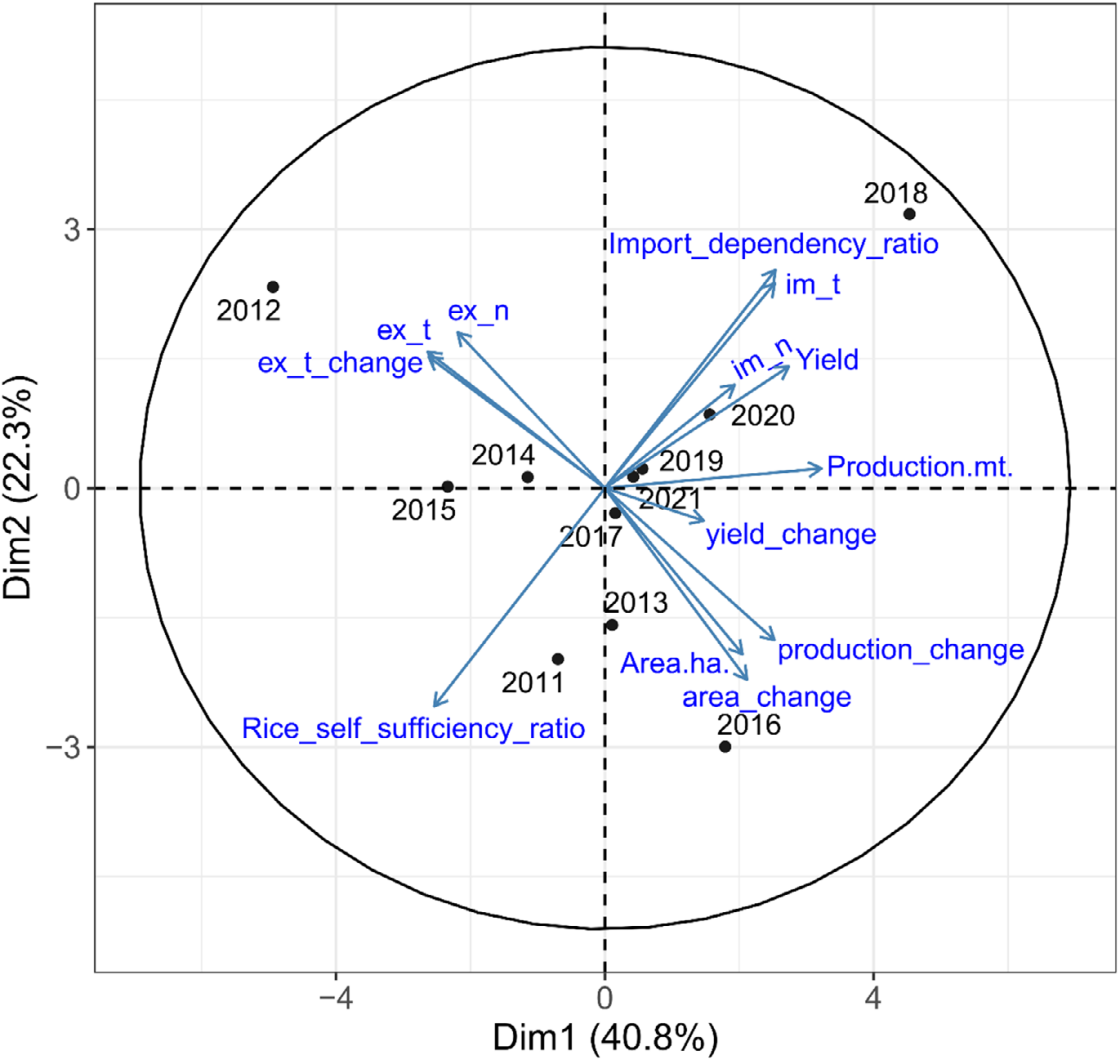
Principal component analysis. Ex_n, export value in Nepali rupees; ex_t, export quantity in metric tons; ex_t_change, rate of change in export in relation to the previous year; im_n, import value in Nepali rupees; im_t, import quantity in metric tons; im_t_change, rate of change in export in relation to the previous year. Fiscal years are represented by the starting year; for example, 2011/2012 is shown as 2011.

Principal component analysis (PCA) was used to display overall patterns in the rice data, and it explained 63.1% of the overall variance in the data. The analysis demonstrated a negative correlation between IDR and RSSR (Figure 4). Notably, the fiscal year 2011/2012 showed high RSSR, while 2018/2019 exhibited a high IDR (Figure 4). The results indicate that the IDR increases with increase in the imports, as expected. However, an increase in the exports does not necessarily correspond to a high RSSR.

### 3.4 Population Dynamics

Nepal's population grew gradually from 2011 to 2021 by 10.07%, rising from 26.49 million (26,494,504) to 29.16 million (29,164,578). However, the proportion of working‐age adults (15–59 age group) increased at a slightly slower rate of 8.14%, from 56.96% in 2011 to 61.60% in 2021. Additionally, the population living abroad increased by 14%, from approximately 1.92 million (1,921,494) in 2011 to around 2.19 million (2,190,592) in 2022 (Table S2).

## 4 Discussion

Rice holds a significant position in terms of both area and production in Nepalese agriculture and plays a crucial role in ensuring food and nutrition security in Nepal (Dahal et al. 2023). This study indicates that the rice SSR fell to 82.01% in 2021/2022, while the IDR rose to 17.98%. Although these values might not yet indicate an acute crisis, trend analysis reveals a concerning pattern: SSR is declining at a rate of 1.15% annually, while IDR is rising by 5.89% each year. If left unchecked, this trajectory could significantly undermine Nepal's rice food security.

The decline in rice self‐sufficiency and rise in import dependency stems from several factors, primarily a reduction in the cultivation area and stagnation in production growth. Although there was no significant trend in the rice cultivation area, the numerical data suggests an annual decrease in 0.81%. (Table 1). This decrease in the rice cultivation area can be partly attributed to a labor shortage resulting from temporary international migration, which draws agricultural workers away from farming (Nepal, Nepal, and Bluffstone 2023) and the conversion of agricultural land to nonagricultural uses (Choudhary et al. 2022).

Despite government investment in cereal production since the 1960s, there has been slow growth in area, production, and yield (Dahal et al. 2023). While the rice cultivation area saw a significant increase from 1990 to 2000, it has since experienced a gradual decline, likely attributable to a shortage of agricultural labor. This trend aligns with broader demographic shifts: over the past decade, Nepal's population grew by 10.07%, while the active population increased by only 8.14% (Table S2). Notably, the greater matter of concern is the 14% rise in the foreign population which may have a substantial impact on the country's agriculture. Foreign migration of the active population affects various sectors, including labor force participation, social security, and rice production and cultivation (Govinda Bahadur et al. 2022). Addressing these population dynamics will be crucial for policymakers to ensure sustainable development and effective resource management in Nepal (Choudhary et al. 2022).

Nepal's rice sector has shown modest improvements over the past decade but continues to face significant challenges. From 2011 to 2021, rice yield increased form 3.212 to 3.45 mt/ha representing 1.97% annual growth. However, overall rice production has only witnessed a slight rise, from 5,072,248 to 5,130,625 mt, an increase in just 1.5% per annum (Figure 1, Table 1).

Nepal's rice productivity remains low compared with the global average of 4.0 mt/ha, primarily due to poor investment in research and technological development (Gauchan and Pandey 2011). Several factors contribute to this underperformance. The government of Nepal has been ineffective in developing and promoting high‐yielding fine and aromatic rice varieties and hybrid varieties widely to farmers (Gairhe, Gauchan, and Timsina 2021). Initiatives like the Prime Minister Agriculture Modernization Project (PMAMP) and community seed banks, aimed at boosting fine and aromatic rice production, have yet to achieve significant success (Bhandari et al. 2017). Structural issues such as land fragmentation, limited access to inputs, and unequal distribution of technologies have hindered farmers, especially those with small or marginal farms, from reaching their full production potential (Gairhe, Gauchan, and Timsina 2021; Malla et al. 2022). Climate change further complicates the situation, with unseasonal heavy monsoons and flooding negatively impacting rice production and exacerbating food insecurity (Malla et al. 2022). Additionally, migration patterns have influenced rice productivity (Govinda Bahadur et al. 2022). To address these challenges, there is a pressing need to empower female household members and improve access to technology and seeds to enhance productivity (Govinda Bahadur et al. 2022). Nepal's continued reliance on rice imports to meet growing demands underscores the urgency of addressing these multifaceted issues (Choudhary et al. 2022; Timsina et al. 2023).

Nepal's rice sector has undergone significant changes in recent years. During the year from 2011/2012 to 2021/2022, the country's reliance on rice imports increased with the IDR rising from 7.28% to 17.99% (Figure 3). Conversely, the ability of domestic production to meet consumption demand declined, as evidenced by the drop in the rice SSR from 92.72% to 82.01% during the same period. Prasad, Pullabhotla, and Ganesh‐Kumar (2019) identified a substantial gap of 19%–80% between domestic rice production and direct household demand. The import of milled or semimilled rice primarily consists of fine and aromatic varieties, indicating an increasing income level and preferences of Nepalese consumers toward high quality of imported rice (Gairhe, Gauchan, and Timsina 2021). This trend coincides with an overall increase in agricultural consumption over the past two decades, indicative of improved living standards and agricultural productivity (Liu et al. 2023). However, the number of agricultural households and the farm population decreased between 2011 and 2021.

To address these challenges, access to inputs, technology, and market linkages, especially for small and marginal farmers and women, is essential for improving rice production efficiency and achieving food security in Nepal (Dhungana 2022). Given the fragility of Nepal's agricultural environment, enhancing agricultural production capacity through structural adjustments, resource efficiency improvements, better inter‐regional agricultural product flow, and improved international food trade channels is crucial (Liu et al. 2023). Factors such as productivity, technology, and stability play a crucial role in Nepal's rice self‐sufficiency (Timsina et al. 2023). Although the agricultural sector supports a significant portion of the population, particularly in subsistence farming, inefficiencies in rice production systems hinder the achievement of full potential output (Liu et al. 2023). Nepal's rice sector and overall food security face significant challenges that require multifaceted solutions. Efforts to enhance agricultural productivity, improve access to inputs, and promote inclusive growth through cooperative farming and market systems approaches are crucial for advancing Nepal's rice sector and overall food security (Poudel et al. 2020). Furthermore, addressing local food supply imbalances, managing fragile agricultural environments, and developing policies to increase agricultural productivity are essential steps toward achieving food self‐sufficiency and eliminating hunger in Nepal (Liu et al. 2023). By implementing these strategies, Nepal can make substantial progress toward a more resilient and sustainable agricultural system that ensures food security. Failure to implement these strategies could jeopardize Nepal's rice food security as the country continues its trajectory of declining self‐sufficiency and increasing import dependency.

## 5 Conclusion

Rice plays a critical role in Nepalese agriculture, both in terms of area and production, and is vital for ensuring food and nutrition security. However, rice self‐sufficiency has declined to 82.01%, while import dependency has risen to 17.98% in 2021/2022. If the current trend of significantly increasing IDR and decreasing RSSR continues, it could severely threaten Nepal's rice food security. The trend shows no significant expansion in cultivated area, with only modest increase in yield. Despite this, overall production remains insufficient to meet consumption demands. The farm population has also decreased. The nation's rice imports are accelerating, both in volume and price, while exports remain negligible in comparison. This analysis highlights the precarious state of rice food security in Nepal, characterized by below averages global yields and production levels that fail to meet national demand. Moreover, the agricultural workforce has experienced a decline. Meanwhile, the trend analysis further underscores a substantial increase in IDR and a corresponding decline in RSSR. If left unchecked, this trajectory could severely undermine Nepal's rice food security. Urgent interventions are needed to address these imbalances and build a more resilient rice sector for the future.

## Conflicts of Interest

The authors declare no conflicts of interest.

## Supporting information


Table S1.
Table S2.

## Data Availability

All the data are within the manuscript and Supporting Information (Tables S1 and S2).
