# Supplementary material for: Rice Cultivation Area, Demographic Trends, and Trade Dynamics for Food Security in Nepal (2011–2021)
Source: Plant Environ Interact. 2024 Dec 6;5(6):e70020. doi: 10.1002/pei3.70020 (PMC11624010; doi:10.1002/pei3.70020)
Supplement: Supplementary file 1 — Table S1. Table S2. [file PEI3-5-e70020-s001.docx]

Supplementary Table 1. Demographic data

| **Demography** | **Year-2011** | **Year- 2021** | **change%** | **pattern** |
| --- | --- | --- | --- | --- |
| Total population | 26,494,504 | 29,164,578 | 10.07784 | Increasing |
| Working age population (15-59) | 0.57 | 0.62 | 8.146067 | Increasing |
| agriculture households (%) | 71 | 62 | -12.6761 | Decreasing |
| Total number of farm population | 20552543 | 19449755 | -5.3657 | Decreasing |
| Foreign population | 1,921,494 | 2,190,592 | 14.00462 | Increasing |

Supplementary Table 2. Rice status and trend

| year | Area(ha) | Production(mt) | Yield | export_quantity_in_metic_tons | export_values_in_Nepali_rupess_NRs | Import_quanity_in_metic_tons | Import_value_in_nepali_rupees_NRs | area_change | production_change | yield_change | ex_t_change | Import_dependency_ratio | Rice_self_sufficiency_ratio |
| --- | --- | --- | --- | --- | --- | --- | --- | --- | --- | --- | --- | --- | --- |
| 2011 | 1531493 | 5072248 | 3.112 | 0.338 | 47004 | 398482.9 | 9.29E+09 | 0.00 |  |  |  | 7.28 | 92.72 |
| 2012 | 1420570 | 4504503 | 3.17 | 919.252 | 29217522 | 529913.6 | 1.43E+10 | -7.24 | -11.19 | 1.86 | 271868.05 | 10.53 | 89.49 |
| 2013 | 1486951 | 5047047 | 3.39 | 123.152 | 8297305 | 502233.3 | 1.73E+10 | 4.67 | 12.04 | 6.94 | -86.60 | 9.05 | 90.95 |
| 2014 | 1425346 | 4788612 | 3.36 |  |  | 723241 | 2.48E+10 | -4.14 | -5.12 | -0.88 | -100.00 | 13.12 | 86.88 |
| 2015 | 1362908 | 4299079 | 3.15 | 0.65 | 199000 | 539607.9 | 2.28E+10 | -4.38 | -10.22 | -6.25 | -99.47 | 11.15 | 88.85 |
| 2016 | 1552469 | 5230327 | 3.37 | 1.787 | 649000 | 590194.1 | 2.39E+10 | 13.91 | 21.66 | 6.98 | 174.92 | 10.14 | 89.86 |
| 2017 | 1469545 | 5151925 | 3.51 | 1.599 | 309000 | 743389.9 | 2.95E+10 | -5.34 | -1.50 | 4.15 | -10.52 | 12.61 | 87.39 |
| 2018 | 1491744 | 5610011 | 3.76 | 11.898 | 4475000 | 7691511 | 3.26E+10 | 1.51 | 8.89 | 7.12 | 644.09 | 57.82 | 42.18 |
| 2019 | 1458915 | 5550878 | 3.8 | 38.313 | 9437000 | 730894.2 | 3.21E+10 | -2.20 | -1.05 | 1.06 | 222.01 | 11.64 | 88.37 |
| 2020 | 1434740 | 5621710 | 3.82 | 14 | 2839000 | 1330656 | 4.86E+10 | -1.66 | 1.28 | 0.53 | -63.46 | 19.14 | 80.86 |
| 2021 | 1477378 | 5130625 | 3.47 | 2.971 | 300000 | 1125401 | 4.67E+10 | 2.97 | -8.74 | -9.16 | -78.78 | 17.99 | 82.01 |
